# Dietary diversity and associated factors among adolescents in eastern Uganda: a cross-sectional study

**DOI:** 10.1186/s12889-020-08669-7

**Published:** 2020-04-19

**Authors:** Nathan Isabirye, Justine Nnakate Bukenya, Mary Nakafeero, Tonny Ssekamatte, David Guwatudde, Wafaie Fawzi

**Affiliations:** 1 School of Public Health, Makerere University, P.O. Box 7072, Kampala, Uganda; 2 Department of Global Health and Population, Harvard University, Boston, USA

**Keywords:** Dietary diversity, Parenting, Food intake, Adolescents, Food groupings, Rural households, Micronutrients

## Abstract

**Background:**

Globally adolescents constitute over 16% but in SSA, they make up 23% of the population. While little is known about diets of these adolescents, rapid changes in physiological and social processes undergone require adequate diets. This study aimed to determine dietary diversity and associated factors among adolescents residing in the Iganga -Mayuge HDSS.

**Methods:**

As part of the African Research, Implementation Science, and Education (ARISE) Network, we analysed collected data among 598 adolescents to assess the health status and adolescents’ behaviour. Dietary diversity was scored using the 9 food group categories as per the Food and Agriculture Organization -WDDS. Crude and adjusted prevalence rate ratios were estimated using the modified Poisson regression model to identify associated factors.

**Results:**

Among the participants, 45.3% had a low dietary diversity score. Proportions of adolescents who consumed from the different food categories over a 24-h period were; cereals/roots/tubers (99.7%), fats & oils (87.0%), spices & beverages (84.1%), sweets (77.1%), legumes (66.2%), other non-vitamin A-rich vegetables (53.8%), dark green leafy vegetables (42.3%), meat/poultry/fish (33.1%), dairy products (32.9%), eggs (11.2%), vitamin A-rich fruits and vegetables (33.4%) and other fruits (8.2%). Staying with a single parent or guardian, low socio-economic class, and dependency on home meals was associated with low dietary diversity.

**Conclusions:**

Adolescents diets were low in diversity and characterised with low micronutrients source foods, but plenty of fats and oils. Interventions to address contributing factors to the burden ought to target the parenting contexts of the adolescents residing in rural eastern Uganda.

## Background

Globally adolescents constitute over 16% (1.2 billion) of the population, but in Sub Saharan Africa (SSA) they make up 23% [1]. Adolescents undergo enormous physiological (rapid growth changes during puberty such as physical activity lean tissue accretion) and social processes (cultural and gender norms; acceptable work types, free time activities, early marriages, and physical activities; changes in access to processed & unhealthy food markets; food supply deficits at household level) that require an adequate and diverse diet [2] to support the growth spurt and normal pubertal development [3]. Despite the low attention given to adolescents, there is an increase in nutritional demand during this period [4]. The majority of nutrition intervention focus on children and mothers and adolescents’ demands are not prioritized [2]. Yet, nutrition remains the leading risk factor contributing to the predominant causes of adolescents and adult morbidity and mortality [2, 5].

Dietary diversity provides insights into household access to a variety of foods and can be used as a proxy for nutrient adequacy of the diet of individuals [6]. An increase in individual dietary diversity score is related to increased nutrient adequacy of the diet [6]. Eating healthy consists of a balanced and varied diet that constitutes; fresh and natural foods, fruits and vegetables and foods containing vitamins and minerals [7]. It also includes practices of good eating behaviours and habits that contribute to physical and psychological wellbeing [8]. However, diets during adolescence are compromised by low socioeconomic status and food insecurity [9].

While adolescents nutrition is given less priority, 1/3 of the adolescents in the world are obese and more than 10% in low and middle income countries are underweight [10]. There is a dearth of information on the burden of nutrient adequacy among adolescents in Uganda. A study conducted among adolescent girls indicated a risk of insufficient intake of multiple micronutrients, especially for vitamins A, B12, C, D, E and calcium, and a low intake of essential fatty acids. In addition, stunting and overweight rates stood at 18.6% and low consumption of animal products such as meat and milk in diet was noted [11]. Paying attention to the nutritional requirements of adolescents is vital in ensuring healthy and productive lives. Adequate nutrition through diverse diets provides an opportunity to correct the deficits suffered during childhood, provide adequate stores of energy for illnesses and pregnancy, and prevents chronic diseases [8, 12, 13] and adult-onset of nutrition-related diseases and disorders such as osteoporosis and obesity [14]. A balanced nutrient intake provided during this critical period of rapid growth [3] has the potential to reduce morbidity and mortality associated with pregnancy and delivery [15–17], and reduces the risk of delivering low birth-weight babies, consequently preventing an intergenerational cycle of malnutrition [9, 14]. Adequate nutrition has also shown a positive impact on adolescents’ education capacity [18]. It is recommended that adolescents should eat from a variety of foods (low fat animal products, fruits, vegetable, grains, staples), drink plenty of water, and should avoid high consumption of sugars.

While studies conducted in other countries give insights to the burden of malnutrition among adolescents, there is limited evidence on diet diversity and associated factors in Uganda [16–24]. Yet, understanding diet diversity across this age range is vital in the appropriate implementation of targeted nutrition interventions [25]. Makerere University being part of the African Research, Implementation Science, and Education (ARISE) Network, enrolled participants residing in their established surveillance site (Iganga Mayuge Health and Demographic Surveillance Site (IMHDSS) to assess health status and behaviour. Specifically, this analysis describes dietary diversity among adolescents and associated factors in two rural districts of Iganga and Mayuge in Eastern Uganda to inform the designing of interventions that promote adolescents’ health.

## Methods

Using a cross-sectional study design, data on dietary diversity was collected during the assessment of health status and behaviour of adolescents residing in Iganga and Mayuge districts. The study utilized the existing IMHDSS data to identify adolescents/households that were enrolled in the survey. Adolescents aged 10–19 by the time of the survey and had resided in IMHDSS for at least one year were considered eligible. A pre-tested questionnaire was used to collect data on socio-demographics, food consumption, and other possible risk factors among 598 adolescents.

### Sampling procedure and measurements

A sample size of 598 adolescents was predetermined based on resources available to conduct the primary study. Adolescents were randomly selected from the IMHDSS database, but stratified in the following two proportions: 80% from the rural parts, and 20% from the townships (peri-urban) of the IMHDSS. Field staffs then used the generated list to visit the households of the adolescents, where they identified the listed adolescents. In case the listed adolescent could not be contacted, traced, or declined to participate in the study, replacement with another adolescent from the nearest household was made. Detailed questionnaires were administered by trained research assistants during a face-to-face interview with participants to obtain socio-demographic data that included: age, parity, education, residence, employment status, socio-economic status variables, marital status, and past/current use of tobacco, alcohol and other substances. The study adapted and validated the MDDW -questionnaire to contain a list of food groups commonly consumed in eastern Uganda. The lists of food groups were developed using the validated food frequency questionnaire used in the previous surveys to assess effect of standard dose multivitamin supplementation on disease progression in HIV-infected adults conducted in Uganda. This tool was translated into local language, pretested and changes were made to ensure consistency and accuracy during data collection process. The respondents were asked to recall the food and beverages consumed from the time of waking until the respondent went to sleep the past day. This helped to indicate on the food list the foods they consumed as appropriate.

### Consenting for participation

A team of consultants made a pre-visit to all IMDHSS before the actual data collection to introduce the study and also to seek permission from local authorities for security reasons. While in the field, informed consent was obtained from all the legal guardians. Upon acceptance of the guardian, assent for adolescents aged less than 18 years was obtained separately. Those above 18 years informed consent was obtained from participants, themselves. Confidentiality was ensured for all study participants by conducting interviews in places with privacy and not using their names for data analysis. We ensured that study participants obtained information about the study, the risks and benefits, and emphasis on the protection of confidentiality. In addition to the informed consent process, an information sheet was given to each respondent. It also included information about key points of contact and phone numbers.

### Quality control

The research assistants were trained before data collection to ensure a mutual understanding of the research protocol as well as the study tools. Research assistants were trained using translated tools to ensure that the survey questions are well understood by both the data collectors and respondents. The study tools were pretested to verify that meaning was not lost during translation as well as to estimate the time required to complete the interview tool.

### Data entry and analysis

Data from the study tools were double entered using Epi Data by independent data clerks to ensure an error-free dataset. STATA SE 14 version was used to analyse the data and to make inferences. Individual Dietary Diversity Score (DDS) was defined as the number of food groups consumed over a 24-h period. The aggregation of foods included in the questionnaire into 9 groups was done according to Food and Agriculture Organisation (FAO) guidelines [26] to create the Women Dietary Diversity Score (WDDS) [27]. Dietary diversity was scored using the 9 food group categories and a cut-off score of ≥4 was considered appropriate (highest sensitivity and specificity) [28, 29] hence was used as the minimum cut off point for having a high dietary diversity during analysis. Crude and adjusted prevalence rate ratios (PRR) were estimated using the modified poisson regression model to identify associated factors. The backward stepwise model selection procedure was used to maintain only variables that were significantly associated with the dietary diversity score of ≥4 (outcome of the study) at a 5% level of significance. The cofounders included in the analysis included; age, sex and socio-economic status of the study participants.

## Results

Data collected from 598 adolescents that participated in the survey were analysed of whom, 52% were male adolescents and 87.3% were still in school. As 88.5% reported both their parents being alive, 70.5% were living with both parents and 18.1% had ever engaged in informal employment. Details are illustrated in Table 1.

**Table 1:** Socio demographic characteristics

Characteristic	Frequency (n) *N* = 598	Percentage %
Gender		
Male	312	52.2
Female	286	47.8
Age (years)		
10–14	321	53.7
15–19	277	46.3
Highest education level presently		
Primary or lower	419	70.1
Secondary or higher	179	29.9
Living status of parents		
Both alive	527	88.5
Single parent or both not alive	71	11.4
Person adolescent lives with		
Both parents	422	70.5
Single parents	117	19.6
Guardian/self	59	9.9
Gainfully employed in the last 12 months		
No	490	81.9
Yes	108	18.1
Social Economical Status (SES)		
Poorest	184	30.8
Poor	104	17.4
Middle class	103	17.2
Rich	90	15.0
Richest	117	19.6
Eat from restaurant		
Never	510	85.3
At least once a week	88	14.7
Household size		
Up to 5 people	116	19.4
6–10 people	391	65.4
More than 10	91	15.2

Low dietary diversity among participants stood at 45.32%. Regarding consumption of an item from the food groups in the previous 24 h, the majority (99.7%) of adolescents reported having consumed cereals/roots/tubers, (87.0%) fats & oils, (84.1%) spices & beverages, and 77.1% consumed sweets. Among the study participants, pulses, nuts, seeds (legumes), and other vegetables (non-vitamin A rich), were averagely consumed ranging from 50 to 66.2%. The least consumed foods included; dark green leafy vegetables (42.3%), meat/poultry/fish (33.1%), dairy products (32.9%), eggs (11.2%), vitamin A-rich fruits and vegetables (33.4%) and other fruits (8.2%). Details are illustrated in Table 2.

**Table 2:** Adolescents intakes presented by food groups and dietary diversity

Food type	Consumed *N* = 598 (n)	Percentage that Consumed (%)
**Grain, white roots and tuber, & plantains**	596	99.7
**Pulses, nuts and seeds**	396	66.2
**Diary**	197	32.9
**Meat, poultry and fish**	198	33.1
**Eggs**	67	11.2
**Dark green leafy vegetable**	253	42.3
**Vitamin A-rich fruits and vegetables**	200	33.4
**Other vegetables**	322	53.8
**Other fruit**	49	8.2
**Oils and fats**	520	87.0
**Sweets**	461	77.1
**Spices and beverages**	503	84.1
**Low dietary diversity (LDD) (< 4 food groups)**	271	45.3

Note: Dietary diversity was scored using the 9 food group categories and a cut off of < 4 was considered as LDD

In the multivariable model, adolescents staying with a single parent and those staying with a guardian or by themselves were more likely to consume a less diverse diet compared to those staying with both parents (aPRRs = 1.31,1.39 Vs aPRR 1.0; 95%CI). Adolescents living in households of the lowest socio-economic class were more likely to have a low dietary diversity score compared to those in higher social-economic classes (aPRRs 0.58,0.74,0.58 Vs aPRR 1.0; 95%CI). Adolescents who ate from a restaurant at least once a week were 40% more likely to have a high dietary diversity score compared to those who depended on household meals. Age, education, employment status, and household size were non-significant factors in determining dietary diversity among these adolescents. Details are illustrated in Tables 3 & 4.

**Table 3:** Dietary diversity among adolescents in the IMHDSS

Characteristic	*N* = 598	dietary diversity range n (row %)		
		< 4	≥4	*p*-value
**Gender**				
Male	312	144 (53.1)	168 (51.4)	0.668
Female	286	127 (46.9)	159 (48.6)	
**Age (years)**				
10–14	321	156 (57.6)	165 (50.5)	0.083
15–19	277	115 (42.4)	162 (49.5)	
**Highest education level attained so far**				
Primary (1–7 years)	419	194 (71.6)	225 (68.8)	0.46
Secondary (> 7 years)	179	77 (28.4)	102 (31.2)	
**Person adolescent lives with**				
Both parents	422	173 (63.8)	249 (76.1)	0.004*
Single parents	117	64 (23.6)	53 (16.2)	
Guardian	59	34 (12.5)	25 (7.6)	
**Gainfully employed in the last 12 months**				
No	490	221 (81.5)	269 (82.3)	0.821
Yes	108	50 (18.5)	58 (17.7)	
**Socio- economic status**				
Poorest	184	114 (42.1)	70 (21.4)	< 0.001*
Poor	104	42 (15.5)	62 (19)	
Middle	103	36 (13.3)	67 (20.5)	
Rich	90	39 (14.4)	51 (15.6)	
Richest	117	40 (14.8)	77 (23.5)	
**Eat from restaurant**				
Never	510	244 (90.0)	266 (81.3)	0.003*
Atleast once a week	88	27 (10.0)	61 (18.7)	
**Household size**				
Up to 5 people	116	58 (21.4)	58 (17.7)	0.485
6–10 people	391	171 (63.1)	220 (67.3)	
More than 10	91	42 (15.5)	49 (15.0)	

*5% level of significance

Note: 9 food categories were considered as recommended by FAO. Individual dietary diversity is categorized as “low dds” if less than 4 food groups consumed, and as “diverse” if ≥4

**Table 4:** Factors associated with dietary intake among adolescents in IMHDSS

		Number with	Unadjusted	Adjusted
	**N (%)**	**Low dietary diversity n(%)**	**PRR (95% CI)**	**PRR (95% CI)**
**Gender**				
**Male**	312	168 (51.4)	1.00	1.00
**Female**	286	159 (48.6)	0.96 (0.81–1.15)	0.96 (0.79–1.14)
**Age (Years)**				
**10–14**	321	165 (50.5)	1.00	1.00
**15–19**	277	162 (49.5)	0.85 (0.71–1.02)	0.96 (0.79–1.15)
**Highest education level attained so far**				
**Primary (1–7 years)**	419	225 (68.8)	1.00	1.00
**Secondary (> 7 years)**	179	102 (31.2)	0.93 (0.76–1.13)	1.10 (0.87–1.39)
**Person adolescent lives with**				
**Both parents**	422	249 (76.1)	1.00	1.00
**Single parents**	117	53 (16.2)	1.33 (1.09–1.63)	1.31 (1.08–1.59)*
**Guardian**	59	25 (7.6)	1.41 (1.1–1.8)	1.39 (1.09–1.76)*
**Gainfully employed in the last 12 months**				
**No**	490	269 (82.3)	1.00	1.00
**Yes**	108	58 (17.7)	1.03 (0.82–1.29)	1.09 (0.85–1.39)
**Socio-economic status (SES)**				
**Poorest**	184	70 (21.4)	1.00	1.00
**Poor**	104	62 (19)	0.65 (0.5–0.85)	0.66 (0.51–0.85)*
**Middle**	103	67 (20.5)	0.56 (0.42–0.75)	0.58 (0.43–0.77)*
**Rich**	90	51 (15.6)	0.7 (0.54–0.91)	0.74 (0.57–0.97)*
**Richest**	117	77 (23.5)	0.55 (0.42–0.73)	0.58 (0.45–0.77)*
**Eat from restaurant**				
**Never**	510	266 (81.3)	1.00	1.00
**Atleast once a week**	88	61 (18.7)	0.64 (0.46–0.89)	0.59 (0.44–0.83)*
**Household size**				
**Up to 5 people**	116	58 (17.7)	1.00	1.00
**6–10 people**	391	220 (67.3)	0.87 (0.71–1.08)	1.04 (0.84–1.28)
**More than 10**	91	49 (15.0)	0.92 (0.69–1.23)	1.21 (0.89–1.62)

*5% level of significance

Note: Sex, age and SES maintained as potential universal confounders

## Discussion

Based on the findings, a considerable proportion of adolescents residing in the IMHDSS had a low dietary diversity. The majority of the adolescents reported consuming high proportions of fats and oils, cereals and low intakes of micronutrient sources (vegetables and fruits). The contributing factors to low diversity among the study participants included; staying with a single parent or guardian, low socio-economic status, and dependency on household meals. Age, gender, education level, employment status, and household size were non-significant. This region being majorly rural, low socio-economic status may be of influence to adolescents’ low diet diversity.

Similar findings were reported by Ochola et al. in a systematic review which observed limited dietary diversity mainly comprising of plant-based food source, with limited animal foods, fruits and vegetable intakes among adolescents residing in rural communities [18, 19]. The majority (83%) of the IMHDSS is rural and the residents in the area are mostly subsistence farmers, whose diets are predominantly plant-based [30, 31]. The prevalence of low diet diversity has an implication on nutritional status and is a pointer to the reducing quality of diets in the IMHDSS. The findings may be explained by the poor farming methods which focus on a few crops in the Mayuge and Iganga districts [30].

A significant proportion of adolescents reported high consumption of fats/oils and beverages; with low intakes of animal source foods, micronutrient source fruits and vegetables. Similar findings were observed by Adam et al., which showed a marked positive relationship between low-income population and increased intake of fats and sweetened beverages. Majority of residents being rural, we still observe a shift in diet and this may be partly explained by the increase in the availability of cheap oils and fats on low-income state markets [32, 33]. Moreover, in Uganda, oils and sweeteners are packed in small quantities increasing availability and affordability.

The study revealed the existence of factors that affect adolescents differently and often relate to the unequal distribution of dietary intakes as observed in other studies [18, 19]. Single parenting in households where these adolescents reside was associated with a low dietary diversity score. Similar findings have been observed in studies done on the relationship between parenting and dietary intake among children [34–36]. Adolescents need extra support to avoid low- quality diets, yet single parents in a resource-poor setting are usually socially and economically constrained [37]. This involves persistent strains such as economic hardship, loneliness, single parenting hardships, reduced on-farm workforce and may explain the negative impact on dietary diversity among adolescents staying with single parents or those who lost one or both parents.

Another important finding was the positive association of low dietary diversity and being a member of a low socio-economic class household. This is similar to findings from several studies previously conducted in LMICs, which reported low socio-economic status as being associated to poor diets and nutritional status of adolescents [38–40]. What may contribute to low dietary diversity in these households is the dependency on mainly home-grown staples and legumes with limitation in access to other important healthy foods which are available in the local markets.

The study revealed a positive association between eating away from home in a restaurant and dietary diversity. This is in contrast to the previous studies that indicated low diverse diets among adolescents who consumed foods away from home [41, 42]. This variation may be explained by the difference in the food types and mode of preparations between fast-foods and the local restaurants found in the IMHDSS. It turns out an opportunity to consume a variety of foods from restaurants which prepare a variety of foods compared to the home meals which are constrained to only foods grown in the home backyard.

### Study limitation

The self-report using a food list used to collect data can be biased for true dietary intakes and may not reflect the routine diet pattern of participants. However, the use of the food lists is a good technique of estimating diversity in diets of individuals other than macro/micronutrient intake estimates for detailed analysis in diets of individuals. Having a detailed estimate of food intake useful in assessing nutrient intakes contained in the diets of individuals. Besides, research assistants were also trained before data collection to ensure consistency and accuracy during the entire data collection process. They were further trained on using illustrations/aiding objects to assist participants in reporting of food types consumed by an individual. At each end of the day, supervisors were assigned to review tools for accuracy and completeness of data collection and this helped in the correction of errors.

## Conclusion

Adolescents residing in rural eastern Uganda are at risk of having low dietary diversity. Their diets are characterized by high consumption of fats and oils but with low intake of micronutrient-rich foods such as fruits and vegetables. This type of diet is nutritionally inadequate, and may perpetuate the double burden of malnutrition among adolescents. Nutrition-related intervention, ought to target the parenting contexts of adolescents. Further studies to understand the quantities and nutrients contained in the diets of these adolescents are recommended.

## Data Availability

The datasets used and/or analysed during the current study are available from the corresponding author on reasonable request.

## Abbreviations

IMHDSS: Iganga Mayuge health and demographic surveillance site
WDDS: Women’s dietary diversity score
DDS: Dietary diversity score
PRR: Prevalence rate ratios
FAO: Food and agriculture organization
LMICs: Low and middle-income countries
ARISE: Africa research, implementation science, and education network
SES: Socio-economic status
